# Supplementary material for: Exact integer linear programming solvers outperform simulated annealing for solving conservation planning problems
Source: PeerJ. 2020 May 27;8:e9258. doi: 10.7717/peerj.9258 (PMC7261139; doi:10.7717/peerj.9258)
Supplement: Supplemental Information 1 [file peerj-08-9258-s001.pdf]

Supporting Information for

Integer Linear programming outperforms simulated annealing for solving conservation planning problems

Richard Schuster, Jeffrey O. Hanson, Matt Strimas-Mackey, Joseph R. Bennett

Appendix S1

Marxan Terminology

Description of some terms used in Marxan analysis. Text marginally modified from the Marxan Manual (v1.8.2): Ball, I. R., & Possingham, H. P. (2000). MARXAN (V1. 8.2). Marine Reserve Design Using Spatially Explicit Annealing, a Manual.

Calibration The objective of calibration is to ensure that the set of solutions Marxan produces are close to the "lowest cost" or optimum. Common user settings to explore in calibration are setting the "Species Penalty Factor", "Number of Iterations", and "Boundary Length Modifier". Those user settings, however, can have a large impact on solution efficiency (Fischer and Church, 2005).

Fischer, D. T., & Church, R. L. (2005). The SITES reserve selection system: a critical review. Environmental Modeling & Assessment, 10(3), 215-228.

Species Penalty Functions The Penalty component of the Marxan objective function is the penalty given to a reserve system for not adequately representing conservation features. It is based on the principle that if a conservation feature is below its target representation level, then the penalty should be an approximation of the cost of raising that conservation feature up to its target representation level.

Number of Iterations The number of iterations set has a substantial bearing on how long each run takes. In general, the number of iterations determines how close Marxan gets to the optimal solution (or at least a very good solution). The number should start high (e.g. 1000000) and then be increased (e.g. 10 million or more is commonly applied on large scale datasets) until there is no substantial

improvement in score as iterations continues to increase. At some point, the extra time required by a higher number of iterations will be better spent doing more runs than spending a long time on each run. Choose an acceptable trade-off between solution efficiency (score, or number of planning units) and execution time (number of iterations).

Boundary Length Modifiers The variable, "BLM" (Boundary Length Modifier), is used to determine how much emphasis should be placed on minimising the overall reserve system boundary length. Minimising this length will produce a more compact reserve system, which may be desirable for a variety of pragmatic reasons. Emphasising the importance of a compact network will mean that your targets are likely to be met in a smaller number of large reserves, generally resulting in an overall larger and more expensive reserve system. Thus, the BLM works counter to the other major goal of Marxan, to minimise the overall cost of the solution. BLM can be thought of as a relative sliding scale, ranging from cheaper fragmented solutions (low BLM) to a more compact expensive ones (high BLM). Because this will have a large influence on the final solutions, some work is needed to ensure an appropriate value (or range of values) is found.

Appendix S2

Integer programming formulation

We will begin by recalling fundamental concepts in systematic conservation planning. Conservation features describe the biodiversity units (e.g. species, communities, habitat types) that are used to inform protected area establishment. Planning units describe the candidate areas for protected area establishment (e.g. cadastral units). Each planning unit contains an amount of each feature (e.g. presence/absence, number of individuals). A prioritisation describes a candidate set of planning units selected for protected establishment. Each feature has a representation target indicating the minimum amount of each feature that ideally should be held in the prioritisation (e.g. 50 presences, 200 individuals). Furthermore, prioritisations that are costly to implement are not desirable, and prioritisations that are excessively spatially fragmented are not desirable. Thus we wish to identify a prioritisation that meets the representation targets for all of the conservation features, with minimal acquisition costs and spatial fragmentation.

We will now express these concepts using mathematical notation. Let I denote the set of conservation features (indexed by i), and T_i denote the conservation target for each feature $i \in I$. Let J denote the set of planning units (indexed by j), and C_j denote the cost of establishing planning unit j as a protected area. Let R_{ij} denote the amount of each feature in each planning unit (e.g. presence or absence of each feature in each planning unit). To describe the spatial arrangement of planning units, let E_j denote the total amount of exposed boundary length of each planning unit. Also let L_{jk} denote the total amount of shared boundary length between each planning unit $j \in J$ and $k \in J$ (where j and k are not equal). Furthermore, to describe our aversion to spatial fragmentation, let p

denote a spatial fragmentation penalty value (equivalent to the “boundary length modifier” parameter in the Marxan decision support tool). Higher penalty values indicate a preference for less fragmented prioritisations.

We will consider the following example to explain the spatial E_j and L_{jk} variables in further detail. Imagine three square planning units (P_1, P_2, P_3) that are each 100×100 m in size and arranged left to right in a line. These planning units each have a total amount of exposed boundary length of 400 m (i.e. $E_1 = 400$, $E_2 = 400$, $E_3 = 400$). Additionally, P_1 and P_2 have a shared boundary length of 100 m (i.e. $L_{1,2} = 100$, $L_{2,1} = 100$); P_2 and P_3 have a shared boundary length of 100 m (i.e. $L_{2,3} = 100$, $L_{3,2} = 100$); and P_1 and P_3 have a shared shared boundary length of 0 m (i.e. $L_{1,3} = 0$ and $L_{3,1} = 0$). Note that planning units do not share any boundary lengths with themselves (i.e. $L_{1,1} = 0$, $L_{2,2} = 0$, $L_{3,3} = 0$).

We use the binary decision variables X_j for planning units $j \in J$ (eqn 1a), and Y_{jk} for planning units $j \in J$ and $k \in J$ (eqn 1b).

$$X_j = \begin{cases} 1, & \text{if } j \text{ selected for prioritisation,} \\ 0, & \text{else} \end{cases} \qquad \text{(eqn 1a)}$$

$$Y_{jk} = \begin{cases} 1, & \text{if both } j \text{ and } k \text{ selected for prioritisation,} \\ 0, & \text{else} \end{cases} \qquad \text{(eqn 1b)}$$

The reserve selection problem can be formulated following:

$$\text{minimize} \sum_{j \in J} X_j C_j + \left(\sum_{j \in J} pE_j \right) - \left(0.5 \times \sum_{j \in J} \sum_{k \in J} pY_{jk} L_{jk} \right) \quad \text{(eqn 2a)}$$

$$\text{subject to} \sum_{j}^{J} R_{ij} \geq T_i \qquad \forall i \in I \quad \text{(eqn 2b)}$$

$$Y_{jk} - X_j \leq 0 \qquad \forall j \in J \quad \text{(eqn 2c)}$$

$$Y_{jk} - X_k \leq 0 \qquad \forall k \in J \quad \text{(eqn 2d)}$$

$$Y_{jk} - X_j - X_k \geq -1 \qquad \forall j \in J, k \in K \quad \text{(eqn 2e)}$$

$$X_j \in \{0, 1\} \qquad \forall j \in J \quad \text{(eqn 2f)}$$

$$Y_{jk} \in \{0, 1\} \qquad \forall j \in J, k \in K \quad \text{(eqn 2g)}$$

The objective function (eqn 2a) is the combined cost of establishing the selected planning units as protected areas and the penalized amount of exposed boundary length associated with the selected planning units. Constraints (eqn 2b) ensure that the conservation targets (T_i) are met for all conservation features. Additionally, constraints (eqns 2c–2e) ensure that the Y_{jk} variables are calculated are correctly (as outlined in Beyer *et al.* 2016). Finally, constraints (eqns 2f and 2g) ensure that the decision variables X_j and Y_{jk} contain zeros or ones.

Table S1

Table S1: List of species that were used as features in our analysis.

Species Code	Common Name	Scientific Name
amegfi	American Goldfinch	Spinus tristis
amekes	American Kestrel	Falco sparverius
amerob	American Robin	Turdus migratorius
annhum	Anna's Hummingbird	Calypte anna
baleag	Bald Eagle	Haliaeetus leucocephalus
barswa	Barn Swallow	Hirundo rustica
brdowl	Barred Owl	Strix varia
belkin1	Belted Kingfisher	Megaceryle alcyon
bewwre	Bewick's Wren	Thryomanes bewickii
bnhcow	Brown-headed Cowbird	Molothrus ater
bkhgro	Black-headed Grosbeak	Pheucticus melanocephalus
brebla	Brewer's Blackbird	Euphagus cyanocephalus
brncre	Brown Creeper	Certhia americana
batpig1	Band-tailed Pigeon	Patagioenas fasciata
bushti	Bushtit	Psaltriparus minimus
cangoo	Canada Goose	Branta canadensis
chbchi	Chestnut-backed Chickadee	Poecile rufescens
cedwax	Cedar Waxwing	Bombycilla cedrorum
chispa	Chipping Sparrow	Spizella passerina
coohaw	Cooper's Hawk	Accipiter cooperii
comrav	Common Raven	Corvus corax
amecro	American Crow	Corvus brachyrhynchos
dowwoo	Downy Woodpecker	Dryobates pubescens
eucdov	Eurasian Collared-Dove	Streptopelia decaocto
eursta	European Starling	Sturnus vulgaris
evegro	Evening Grosbeak	Coccothraustes vespertinus
norfli	Northern Flicker	Colaptes auratus
foxspa	Fox Sparrow	Passerella iliaca
gockin	Golden-crowned Kinglet	Regulus satrapa
haiwoo	Hairy Woodpecker	Dryobates villosus
houfin	House Finch	Haemorhous mexicanus
houspa	House Sparrow	Passer domesticus
houwre	House Wren	Troglodytes aedon
hutvir	Hutton's Vireo	Vireo huttoni
macwar	MacGillivray's Warbler	Geothlypis tolmiei
moudov	Mourning Dove	Zenaida macroura
norhar1	Hen Harrier	Circus cyaneus
orcwar	Orange-crowned Warbler	Oreothlypis celata
olsfly	Olive-sided Flycatcher	Contopus cooperi
osprey	Osprey	Pandion haliaetus
pacwre1	Pacific Wren	Troglodytes pacificus
pinsis	Pine Siskin	Spinus pinus
pilwoo	Pileated Woodpecker	Dryocopus pileatus
pasfly	Pacific-slope Flycatcher	Empidonax difficilis
purfin	Purple Finch	Haemorhous purpureus
purmar	Purple Martin	Progne subis
rebnut	Red-breasted Nuthatch	Sitta canadensis
rebsap	Red-breasted Sapsucker	Sphyrapicus ruber
redcro	Red Crossbill	Loxia curvirostra
rocpig	Rock Pigeon	Columba livia
rethaw	Red-tailed Hawk	Buteo jamaicensis
rufhum	Rufous Hummingbird	Selasphorus rufus
rewbla	Red-winged Blackbird	Agelaius phoeniceus
savspa	Savannah Sparrow	Passerculus sandwichensis
sora	Sora	Porzana carolina
sonspa	Song Sparrow	Melospiza melodia
spotow	Spotted Towhee	Pipilo maculatus
stejay	Steller's Jay	Cyanocitta stelleri
swathr	Swainson's Thrush	Catharus ustulatus

Species Code	Common Name	Scientific Name
towwar	Townsend's Warbler	Setophaga townsendi
treswa	Tree Swallow	Tachycineta bicolor
daejun	Dark-eyed Junco	Junco hyemalis
yerwar	Yellow-rumped Warbler	Setophaga coronata
varthr	Varied Thrush	Ixoreus naevius
vigswa	Violet-green Swallow	Tachycineta thalassina
warvir	Warbling Vireo	Vireo gilvus
whcspa	White-crowned Sparrow	Zonotrichia leucophrys
westan	Western Tanager	Piranga ludoviciana
wilsni1	Wilson's Snipe	Gallinago delicata
wlswar	Wilson's Warbler	Cardellina pusilla
wooduc	Wood Duck	Aix sponsa
yelwar	Yellow Warbler	Setophaga petechia

Figure S1

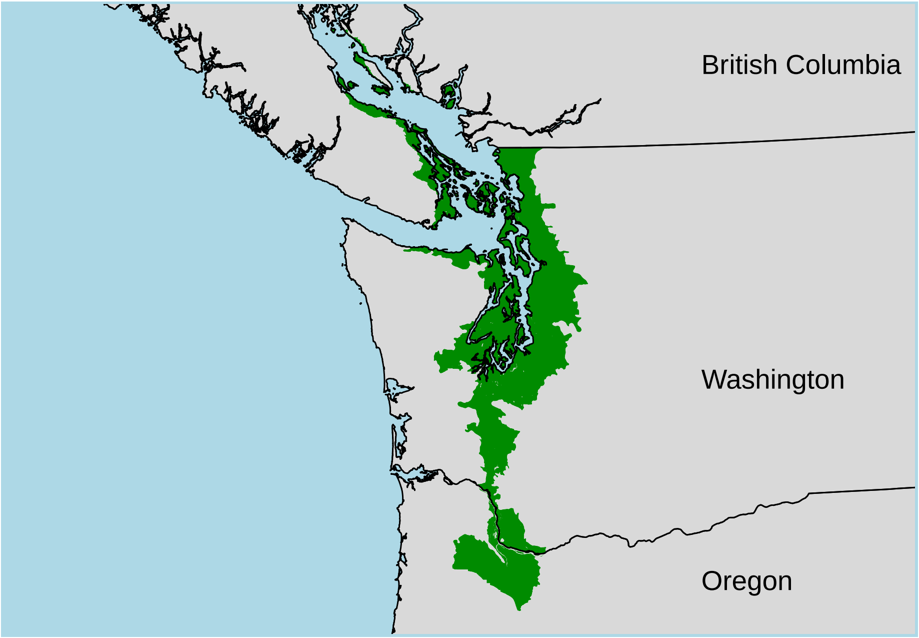

Figure S1: Study area.

Figure S2

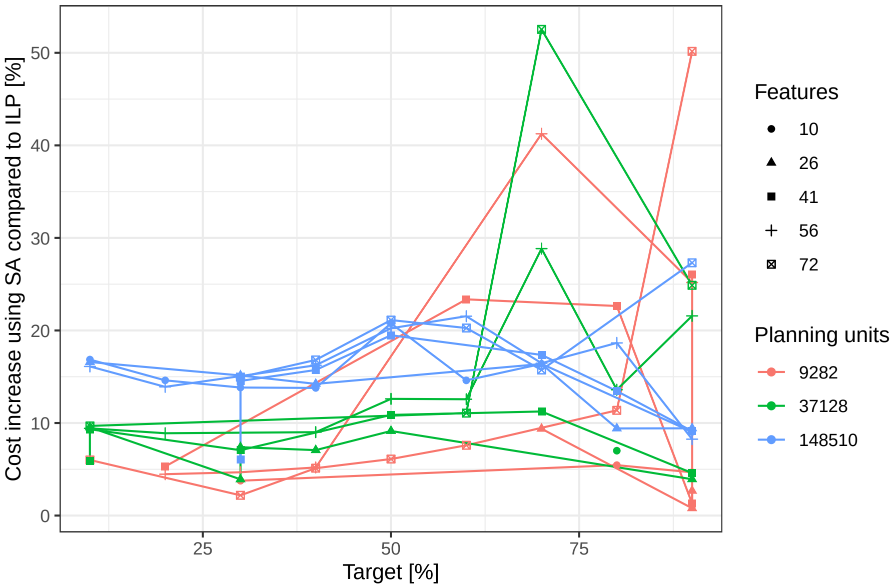

Figure S2: Percent cost increase of SA solutions compared to ILP solutions, across targets, number of features and number of planning units. Simulated annealing (i.e. Marxan) parameters used are: number of iterations > 100,000; species penalty factor 5 or 25. Not all Marxan scenarios generated yielded feasible solutions (where all targets were met), which is why e.g. there is only one observation for 37,128 planning units and 10 features.

Figure S3

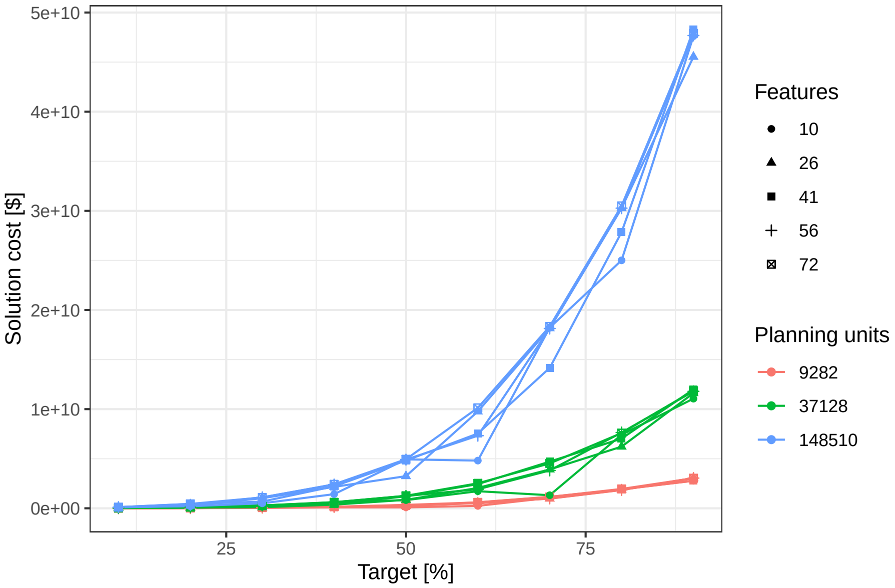

Figure S3: Cost profile for Gurobi solver across targets, number of features and number of planning units.

Figure S4

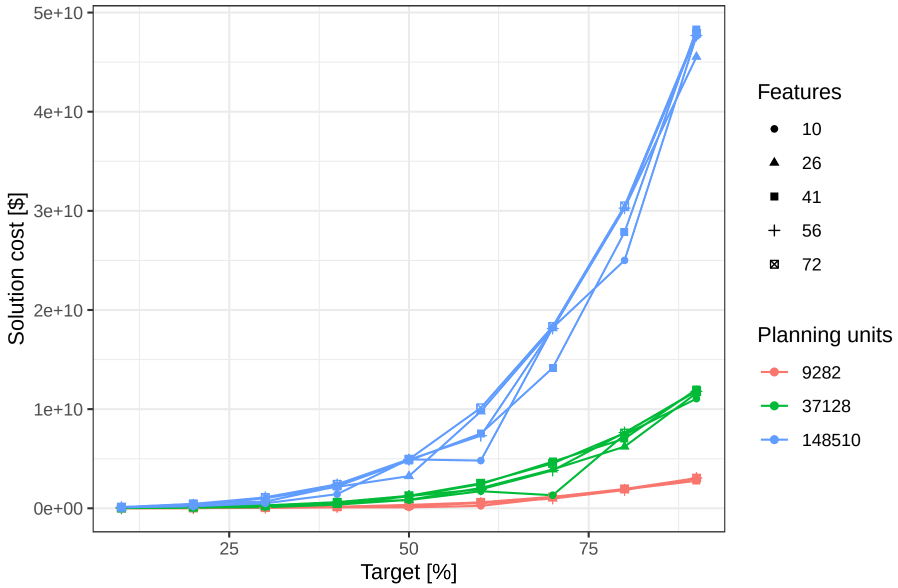

Figure S4: Cost profile for SYMPHONY solver across targets, number of features and number of planning units.

Figure S5

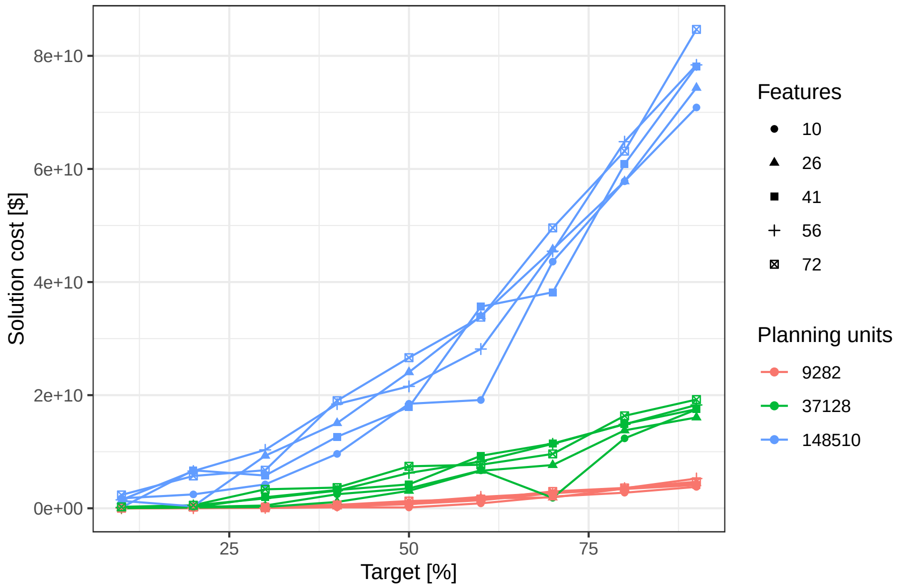

Figure S5: Cost profile for Marxan using Simulated Annealing across targets, number of features and number of planning units.

Figure S6

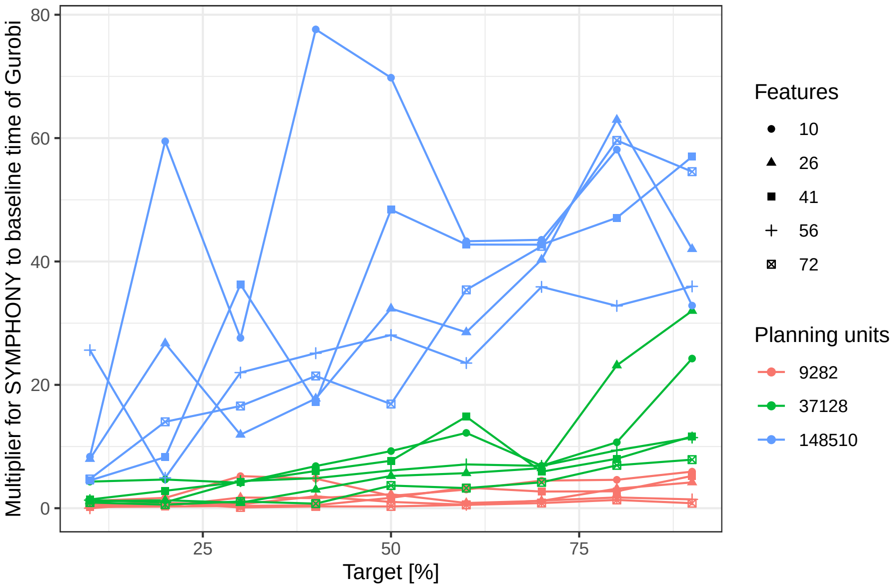

Figure S6: Time to solution comparisons between SYMPHONY and Gurobi across targets, number of features and number of planning units.

Figure S7

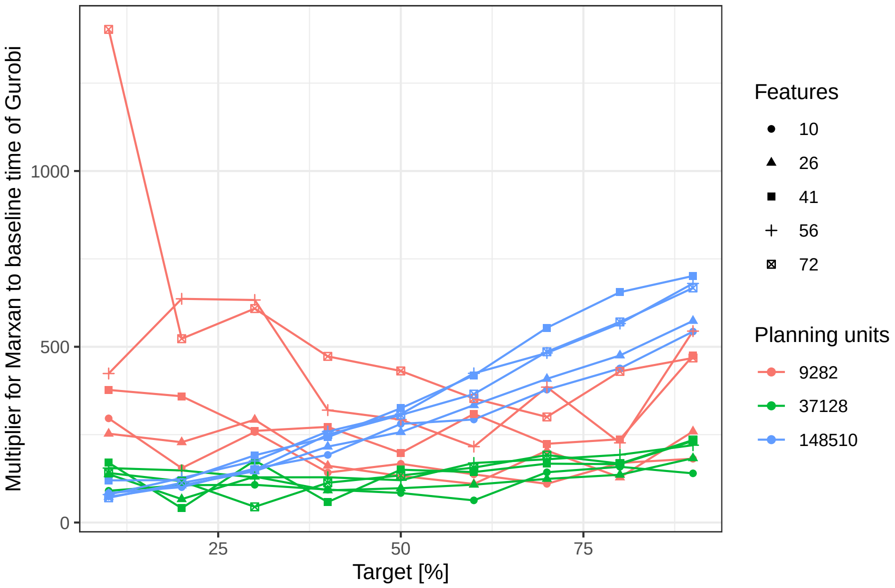

Figure S7: Time to solution comparisons between Marxan using Simulated Annealing and Gurobi across targets, number of features and number of planning units.

Figure S8

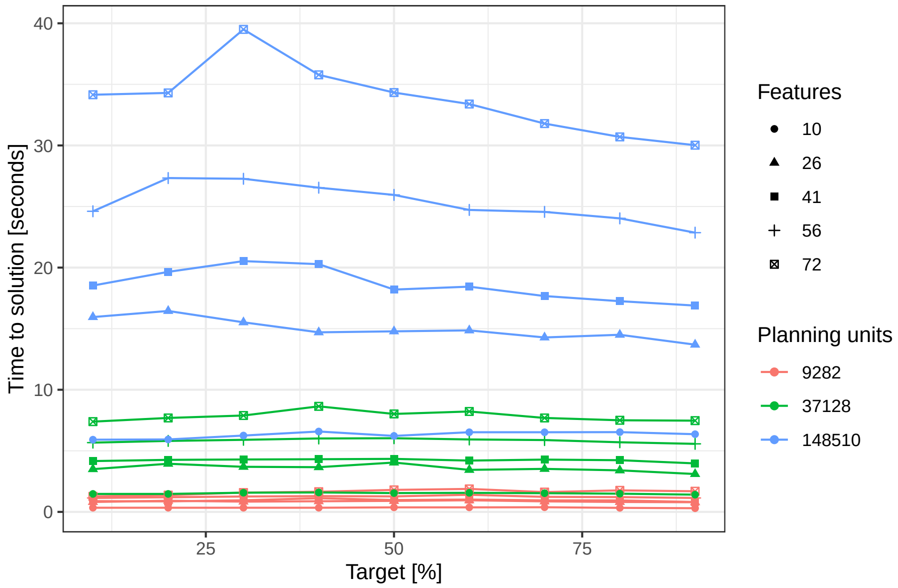

Figure S8: Time to solution profile for Gurobi solver across targets, number of features and number of planning units.

Figure S9

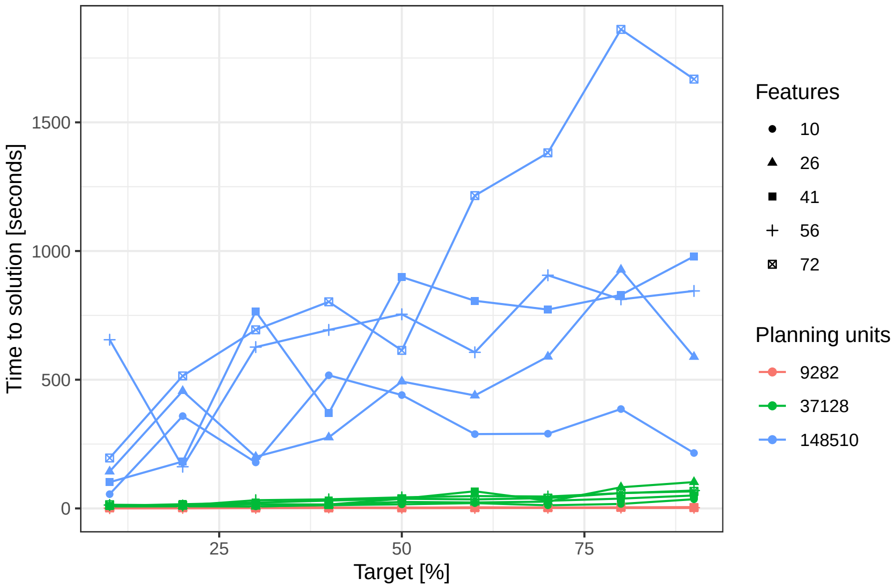

Figure S9: Time to solution profile for SYMPHONY solver across targets, number of features and number of planning units.

Figure S10

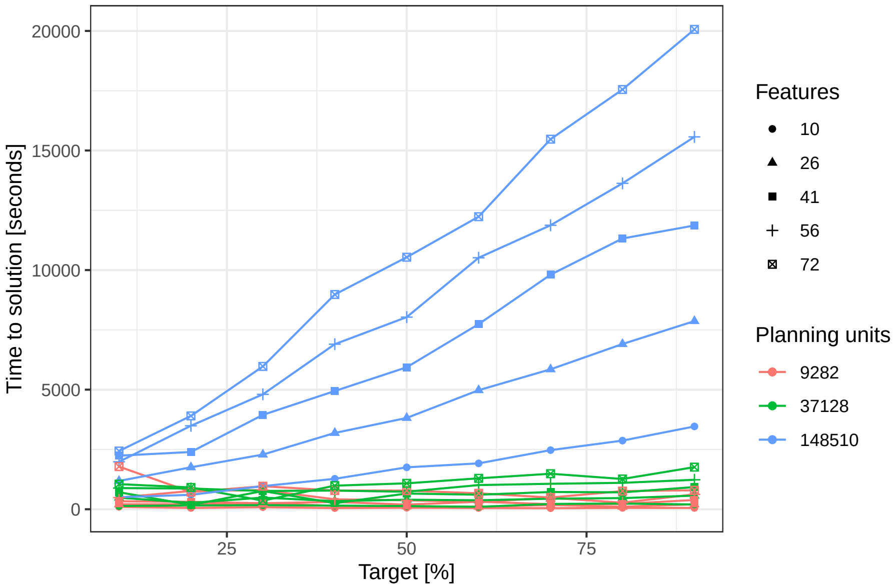

Figure S10: Time to solution profile for Marxan using Simulated Annealing across targets, number of features and number of planning units.

Figure S11

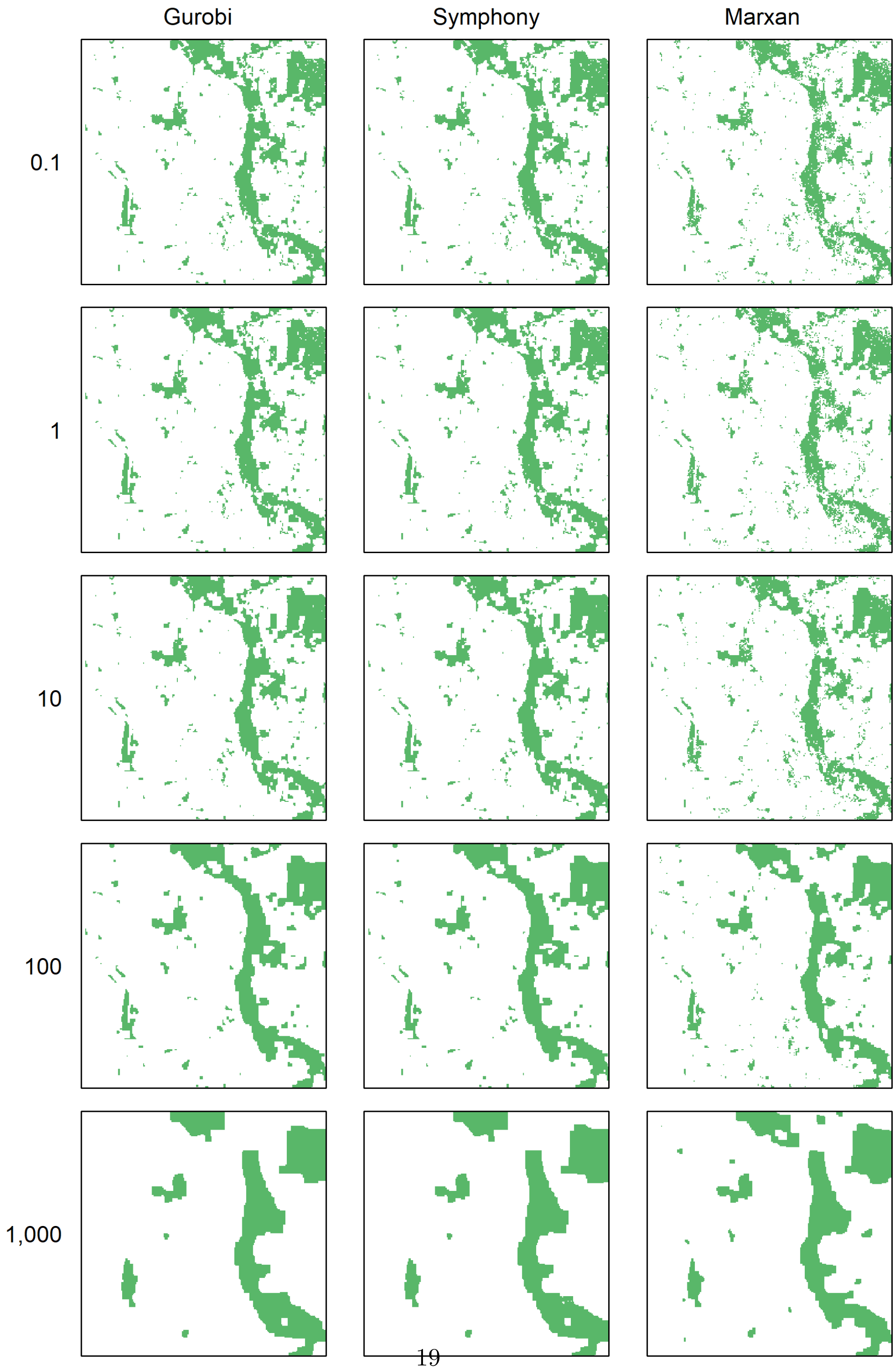

Figure S11: Compactness of solutions. Shown are the solutions for a 10%

target. The numbers represent BLM values.
